# Association between the Nutritional Quality of Household At-Home Food Purchases and Chronic Diseases and Risk Factors in the United States, 2015

**DOI:** 10.3390/nu13093260

**Published:** 2021-09-18

**Authors:** Armen Ghazaryan, Andrea Carlson, Alana Y. Rhone, Kakoli Roy

**Affiliations:** 1 Office of the Director (OD), National Center for Chronic Disease Prevention and Health Promotion (NCCDPHP), Centers for Disease Control and Prevention (CDC), Atlanta, GA 30341, USA; kjr3@cdc.gov; 2 Economic Research Service (ERS), United States Department of Agriculture (USDA), Washington, DC 20024, USA; andrea.carlson@usda.gov (A.C.); alana.y.rhone@usda.gov (A.Y.R.)

**Keywords:** Healthy Eating Index, type 2 diabetes, cardiovascular disease, obesity, smoking, chronic diseases, scanner data, IRI Consumer Network, IRI MedProfiler

## Abstract

Lower diet quality is a leading preventable risk factor for obesity and chronic diseases. This study assesses differences in the nutritional quality of at-home food purchases, using the Healthy Eating Index (HEI)-2015 and its components, among households with and without a member reporting type 2 diabetes (T2D), cardiovascular disease (CVD), obesity, and/or smoking. We use the 2015 IRI Consumer Network nationally representative household food purchase scanner data, combined with the IRI MedProfiler and the USDA’s Purchase-to-Plate Crosswalk datasets. For each/multiple condition(s), the difference in mean HEI score adjusted for covariates is tested for equivalence with the respective score against households without any member with the condition(s). The HEI score is higher for households without a member with reported T2D (2.4% higher), CVD (3.2%), obesity (3.3%), none of the three conditions (6.1%, vs. all three conditions), and smoking (10.5%) than for those with a member with the respective condition. Households with a member with T2D score better on the added sugar component than those with no member reporting T2D. We found that the average food purchase quality is lower than the recommended levels, especially for households with at least one member reporting a chronic condition(s).

## 1. Introduction

Poor diet is a major preventable risk factor for the leading causes of chronic disease in the United States, including obesity, heart disease, diabetes, hyperlipidemia, and stroke [1]. Although nutrition research has traditionally focused on individual dietary components and food groups, an increasing number of studies illustrate the importance of interactive and cumulative effects of eating patterns and diet quality/healthfulness on health outcomes [1,2,3,4]. One such measure of diet quality is the Healthy Eating Index 2015 (HEI-2015), which examines the alignment of dietary patterns with the Dietary Guidelines for Americans 2015–2020 [5] (range 0–100, with 100 suggesting full alignment), with previous iterations of the measure—HEI-2005 and HEI-2010—indicating alignment with the DGA 2005–2010 and DGA 2010–2015, respectively [2].

There is growing literature relating higher HEI scores (among other diet quality measures) to better health outcomes/reduced risk factors, and findings from these studies have been summarized in systematic reviews and meta-analyses [6,7]. To our knowledge, studies analyzing the association between HEI scores and type 2 diabetes (T2D), cardiovascular disease (CVD) incidence and/or mortality, obesity, and smoking status have relied primarily on 24-h dietary recall data [8,9] (e.g., the National Health and Nutrition Examination Survey (NHANES)) and food frequency questionnaire data [10,11,12,13,14,15,16]. The advantages and limitations of constructing HEI scores based on these data have been discussed in the literature [2]. In addition, most of these studies focused on specific population subgroups, such as women in general [10] and postmenopausal women specifically [11], survey participants from lower-income households in the southern U.S. states [14,15], or participants recruited from certain states and communities [3,12,13,16].

The objective of this study is to analyze the difference in the retail food purchase quality (measured in terms of HEI-2015) by household income and among households with and without a member with reported T2D, CVD, obesity, or smoking. We use nationally representative household food purchase scanner data, which, unlike data from dietary recall and food frequency questionnaires, cover food purchases over an entire year. We try to explore two research questions. First, does retail food purchase quality for at-home consumption improve with household income? Second, do households with member(s) with one or multiple chronic conditions/risk factors purchase lower quality retail food for at-home consumption than those without the given health condition? Even though store food purchases do not necessarily translate into intake, they have been shown to produce similar HEI scores as those calculated based on household food acquisition data, dietary intake, and national food availability data [17]. Focusing on quality of household food purchases instead of individual food intake also allows exploring the role that the household food environment plays in shaping individual dietary intake and health outcomes [18,19], which, in turn, may provide insights for intervention strategies to improve dietary quality.

## 2. Materials and Methods

The IRI Consumer Network Panel (CNP) dataset from 2015 provides information on household retail food purchases. The data are derived from the National Consumer Panel (NCP), which is a joint venture owned by IRI and The Nielsen Company [20]. Third-party vendors providing online advertising (e.g., via display networks, blogs, email, social media, etc.) recruit households for the NCP, who can then sign up on NCP’s online recruitment site and answer a detailed questionnaire on household demographics [20]. To make the panel representative of the U.S population in the 48 contiguous states, a random sample of households was selected for membership based on quotas for each type of household [20]. More details about household recruitment and selection are provided in the ERS’s technical bulletin [20]. Selected households recorded all retail food purchases by scanning each product’s bar code throughout the year. Households’ race and ethnicity were self-defined and may represent either the primary respondent’s characteristics, those of other members of the household, or the entire household’s characteristics [20,21]. Using specific thresholds for expenditures based on household size, IRI determines whether a household provides sufficient purchase data and defines them as a static panel of households [20]. We merged the demographic and food purchase data with the IRI MedProfiler 2015, which is a subset of households from the CNP with responses to an annual opt-in survey on health characteristics of household members [20]. All CNP households were offered to participate in the survey and about 52% of the households in the static panel had at least one person respond to the MedProfiler survey in 2015 (73,490 individual respondents). We focused our analysis only on those households participating in both datasets (CNP and MedProfiler) and used the MedProfiler projection factors (survey weights), which indicate how many households are represented by each participating household in the static panel. IRI assigns projection factors to participating households to match the Census targets by weighting the data based on geographic and demographic characteristics.

Participants in the MedProfiler survey were asked to respond to questions regarding common health characteristics with one of five possible responses: suffer but do not treat, suffer and treat with a prescription, suffer and treat with over-the-counter medication, suffer and treat with both prescription and over-the-counter medications, or do not suffer. Based on this information, we created four binary indicators, which were equal to one for households in which anyone reports suffering from one of the following conditions: congestive heart failure, heart attack, high blood pressure, or stroke (henceforth CVD); obesity; T2D; all three conditions combined; and another binary variable indicating whether someone aged 21 or over in the household reported smoking cigarettes.

Next, we combined the merged subsample with the USDA’s Purchase-to-Plate Crosswalk (PPC) dataset, which links IRI scanner data with USDA nutrition databases (nutrient and food group quantities) [17]. Quantities for random-weight products (i.e., products purchased by the pound or unit) are not available in the IRI data [20]. Therefore, PPC does not include nutrition information for these products [17]. As a result, households whose reported purchases consisted of only such random-weight products, for which the quantity and nutrition information was missing, were excluded from the analysis. We used the combined data to calculate household HEI-2015 scores after aggregating all reported food purchases over an entire year at the household level. To compare the average HEI scores and the sub-scores, we first stratified the data by household poverty level, following the U.S. Federal Poverty Guidelines for 2015 [22]: (a) at or below 130% of poverty; (b) between 130% and 185% of poverty; and (c) above 185% of poverty (higher percentage reflecting higher per person household income). The stratification by income was implemented to capture household eligibility for certain programs, such as the Supplemental Nutrition Assistance Program (SNAP) and the National School Lunch Program (NSLP). Then, for each/multiple condition(s)/health behavior(s), the difference in mean HEI score was tested for equivalence with the respective score against households without any member with the condition(s)/behavior(s). We compared the mean HEI scores and its sub-scores adjusted for demographic and socioeconomic factors, such as household poverty level, size, race, and ethnicity; household head’s age and marital status; and whether a household is female-led. We used Stata version 16 (StataCorp LLC, College Station, TX, USA) to perform the analyses and used Stata’s SVY commands to account for the complex sampling design and weighting. The results reported are based on the weighted sample.

## 3. Results

### 3.1. Household Characteristics

Household characteristics, both overall and stratified by income levels, are presented in Table 1. The static household panel for which there were enough nutrition data (i.e., households whose reported purchases do not consist of only random-weight products) to construct HEI scores is composed of 63,578 households. In the data used, 15.2% of the households are at or below the 130% of poverty (henceforth, income group 1 (IG1)); 8.8% are between 130% and 185% of poverty (income group 2 (IG2)); and 76% are above 185% of poverty (income group 3 (IG3)). The average household head’s age is 52.5 with an average household size of almost 3 people. The racial/ethnic composition of the primary respondent for the household in the sample is as follows: 76.8% white, 11.8% black, 4.3% Asian, and 7.1% other; 11.8% identified as Hispanic. About 61.8% of households reported having a married household head(s) and 27.5% having only a female household head. Only female household head means that the household does not have a male household head but it does not necessarily mean that the female is unmarried and/or that it is a single household.

Table 2 presents the percentage of households with a member with one or multiple health outcomes/behaviors. The sample size for households with both health condition/behavior data and enough information to construct HEI scores is 33,179. In our sample, 14.1%, 42.2%, 21.5%, and 16.4% of households have at least one member with reported T2D, CVD, obesity, or smoking, respectively. When stratified by income, the values are higher for households in IG1 and lowest for IG3 for all conditions except for CVD, for which households in IG2 have the highest percent (44.7%), followed by IG3 (42.3%) and IG1 (39.9%). About 4.5% of households reported all three conditions (T2D, CVD, and obesity), and 46.6% of households reported not having any of the three conditions. About 27% and 22.1% of households in IG1 and IG2, respectively, have an adult smoker, while among IG3 households the value is 13.5%.

### 3.2. HEI Scores by Income

Nutrition quality of food purchases, overall and by income, is reported in Table 3. In 2015, the total HEI-2015 score of food purchased by U.S. households was 51.9 (SD 1.8) out of a possible 100. Households in IG1 had the lowest HEI-2015 score in the analysis adjusted for household size, race, and ethnicity; household head’s age and marital status; and whether a household is female-led. The difference in the HEI score between the IG1 and IG2 households is 1.4 points (*p* < 0.001), while that between the IG2 and IG3 households is 3 points (*p* < 0.001).

The HEI-2015 sub-scores illustrate a similar pattern. For most sub-scores, IG3 households have the highest (better quality) scores, followed by IG2 and IG1 households, who have the lowest (poorer quality) scores. All differences are statistically significant (*p* < 0.001). The exceptions are the scores for fatty acid, refined grains, and saturated fats, where IG1 households have higher scores than IG2 households; for refined grains and saturated fats, IG2 households have higher scores than IG3 households. The only statistically insignificant difference is for the total dairy component score between IG2 and IG3 households. The largest relative difference in the sub-scores between IG1 and IG2 households as well as IG2 and IG3 households is in the whole fruit component.

### 3.3. HEI Scores by Health Condition/Behavior

Nutrition quality of food purchases for households with and without a member with reported T2D or CVD is reported in Table 4. The HEI score is 2.4% (*p* < 0.001) higher for households without a member with reported T2D (mean 52.4, SD 2.0) compared to households with a member with T2D (mean 51.2, SD 1.9). When comparing the difference in HEI components, the former has higher scores (better conformance with Federal recommendations) on most components (*p* < 0.001), ranging from 0.4% (seafood and plant proteins) to 17.4% (total fruits). However, households with a member with T2D have higher scores (*p* < 0.001) for the following sub-components: refined grains (by 0.5%), total vegetables (1.1%), total protein foods (3.8%), fatty acid (6.3%), and added sugar (6.8%).

Households without a member with CVD have an HEI score of 52.9 (SD 2.2), which is 3.2% (*p* < 0.001) higher than that for households with at least one member with reported CVD (mean 51.3, SD 1.9). The former group has higher scores for 11 out of 13 components, with the difference ranging between 0.5% (total vegetables, *p* < 0.001) and 14.4% (whole fruits, *p* < 0.001). The only HEI components for which households with a member with CVD have somewhat higher scores are refined grains (higher by 0.4%, *p* < 0.001) and fatty acid (2.7%, *p* < 0.001).

Nutrition quality of food purchases for households with and without a member with reported obesity or the three conditions (T2D, CVD, and obesity) is reported in Table 5. Households with no members with obesity have a higher HEI score (3.3% higher, *p* < 0.001) than those with at least one member with reported obesity. In this case, too, the former group has higher scores for most HEI components than those with a member with obesity, with the difference ranging from 0.5% (seafood and plant proteins, *p* < 0.001) to 14.1% (total fruits, *p* < 0.001). However, the scores for refined grains and total protein foods are higher for households with at least one member with reported obesity by 1.4% (*p* < 0.001) and 2.4% (*p* < 0.001), respectively.

Households with neither of the three health conditions have an HEI score of 53.3 (SD 2.2), which is 6.1% (*p* < 0.001) higher than that for households with member(s) with all three conditions (mean 50.2, SD 2.1). The former group has higher scores for 9 out of 13 components, with the difference in scores ranging from 2.3% (total vegetables (*p* = 0.02) and total dairy (*p* < 0.001)) to 28% (total fruits (*p* < 0.001)). Even though households with member(s) with all three conditions score higher on four components, the difference in those sub-scores is only between 0.4% (fatty acid (*p* < 0.001)) and 2.7% (added sugar (*p* < 0.001)).

Nutrition quality of food purchases for households with and without a member reporting smoking is presented in Table 6. The difference in overall food purchase quality is the largest (10.5%, *p* < 0.001) between households where none of the adults reported smoking (mean HEI score of 52.9, SD 1.8) and those where at least one adult reported smoking (mean 47.9, SD 1.3). Non-smoker households score higher on all except for one component (refined grain) and the difference in sub-scores ranges from 1.5% (fatty acid (*p* < 0.001)) to 59.6% (whole fruits (*p* < 0.001)).

## 4. Discussion

The study analyzed the retail food purchase quality, measured in terms of HEI-2015, for a nationally representative sample of US households and compared the difference in HEI scores and component scores based on household income and reported health conditions/behavior. The findings suggest that higher-income households tend to purchase higher quality food compared to lower-income households, even though the latter score slightly higher on some of the HEI components. While higher-income households have, on average, slightly higher HEI scores, the mean HEI score of 52.6 (SD 1.1) out of 100 for households above 185% of poverty indicates a substantial need for improvement in diet quality to better conform with Federal recommendations for 13 dietary components. These general findings are consistent with evidence from previous studies based on other data sources, such as dietary recall and food frequency questionnaire data [18].

Households with at least one member reporting one of the four chronic conditions/health behaviors have statistically significantly lower HEI-2015 scores than those with no member reporting the condition(s)/behavior(s). A novel finding is that households with a member with T2D score better on the added sugar component than those with no member reporting T2D, which means that the share of purchased calories from added sugars is less for the former. Additionally, the finding that households with a member with T2D score lower on the total fruits and whole fruits components, suggesting that the share of purchased calories from those products are lower, may indicate that these households are avoiding purchasing fruits to avoid natural sugars as well. Our findings indicating lower HEI scores among households with members with chronic conditions/behaviors are consistent with previous studies. For example, using the IRI Consumer Network and MedProfiler datasets with the USDAScore [23] or fruit and vegetable share of food purchases [24] as a measures of food purchase quality, it has been shown that high food purchase quality is negatively associated with obesity status/incidence of obesity. In contrast, using the same datasets and Healthshare score as a measure of food purchase quality, one study [25] found that those with T2D have a higher household food purchase quality than those without T2D.

While food purchases do not necessarily fully translate into food intake, our findings are also consistent with studies analyzing the association between diet quality and chronic diseases. Past studies have found that higher-quality diets are associated with lower incidence and odds of obesity [23,24] and that smokers have lower HEIs than non-smokers [26]. Systematic reviews and meta-analyses [6,27] of cohort studies on health outcomes and diet quality measured in terms of HEI, Alternative HEI (AHEI), and Dietary Approaches to Stop Hypertension (DASH) scores found that higher diet quality is associated with significant risk reduction for all-cause mortality, CVD, cancer, T2D, and neurodegenerative diseases.

### Contributions and Limitations

This study complements the existing literature in two major ways. First, most studies analyzing the association between HEI scores and health outcomes have relied on self-reported 24-h dietary recall data [6,8,9,27] and food frequency questionnaire data [10,11,12,13,14,15,16]. Major limitations of constructing HEI scores based on these data are that scores based on one or a few days of food intake do not necessarily reflect usual individual diet (24-h recall data) and there are systematic biases (food frequency data), due in part to not capturing the entire diet and the difficulty of the recall task [2]. In contrast, this study focuses on retail food purchases over an entire year, which provides a more comprehensive view of household at-home food environments and potentially contains less bias. Second, this study includes multiple health conditions/behaviors individually and combined, whereas past studies, to our knowledge, have primarily focused on one or two conditions at a time regardless of the type of utilized data [6,23,24,25,27].

Our study comes with several limitations. First, our measures of food quality are based solely on food-at-home purchase data, thus omitting the impact of food-away-from-home on diet and the potential associations with household income and health outcomes/behaviors. According to ERS, the normalized food-at-home expenditure by households as a percentage of total food expenditure (food-at-home and food-away-from-home) was 53% in 2015 [28]. Second, given that we use food scanner data, we can only measure the healthfulness of food purchases but not consumption. Even though food purchase does not fully capture food intake [29], it is a proxy of the healthfulness of the home food environments [30]. Third, due to the structure of the data, our measure of food purchase quality and health outcomes/behaviors are at the household-level. Therefore, we are unable to capture individual-level differences and variations, although home food environments do shape the individual dietary intake [19,31]. Fourth, our analysis only focuses on those households participating in both datasets (CNP and MedProfiler). This represents just under half of the static CNP, but the statistical properties, such as comparisons to government-collected data, have not been evaluated [20]. Fifth, the panel HEI scores might be lower because fruits, vegetables, eggs, and seafood tend to be under reported compared to government expenditure surveys. Additionally, since we do not know the quantities of the random-weight fruit and vegetable purchases, about half of the expenditures on produce is omitted from the analysis. However, the impact of excluded random-weight products on HEI is unknown because different product categories (i.e., fresh meat, poultry, seafood, bakery, fruits, vegetables, cheese, cold cuts and lunch meat, prepared foods, coffee, and candy, nuts, and seeds) affect the HEI differently. Lastly, the health outcomes/behaviors are self-reported, meaning that some respondents may not have diagnosed/be aware of their health conditions at the time of reporting, thus being categorized as non-T2D/CVD/obesity households in this study.

## 5. Conclusions

Given that the average food purchase quality is lower than the recommended levels, especially for those with diet-related chronic diseases, the findings from this study suggest the need for greater efforts to promote healthier food purchases among Americans. Strategies identified as effective in systematic reviews [32,33], such as pricing interventions, advertising/promoting healthier food items, monetary nudges, and nutrition counseling, could be implemented to improve food purchase quality and subsequent diet-related chronic conditions.

## Figures and Tables

**Table 1:** Household characteristics, overall and by poverty level, based on Information Resources Inc. (IRI) Consumer Network 2015 data.

	All Incomes	Household (HH) Income < = 130% Poverty Income Group (IG)1	HH Income (130–185%] Poverty IG2	HH Income > 185% Poverty IG3	*n*
Number of observations	63,578	6944	4769	51,865	
Weighted% of households		15.19 (14.79–15.59)	8.81 (8.52–9.10)	76.00 (75.54–76.45)	
	Weighted mean (95% CI)				
White	76.78 (76.33–77.24)	73.84 (72.43–75.26)	77.90 (76.32–79.48)	77.24 (76.75–77.73)	51,544
Black	11.81 (11.49–12.13)	13.64 (12.59–14.68)	11.90 (10.73–13.07)	11.43 (11.09–11.78)	6909
Asian	4.30 (4.08–4.52)	2.90 (2.30–3.49)	2.66 (2.06–3.26)	4.77 (4.51–5.02)	2151
Other	7.11 (6.78–7.43)	9.62 (8.54–10.70)	7.54 (6.39–8.69)	6.55 (6.21–6.90)	2974
Hispanic	11.84 (11.41–12.27)	13.82 (12.48–15.17)	12.98 (11.45–14.51)	11.31 (10.84–11.78)	3988
Married household head	61.77 (61.29–62.25)	45.58 (44.11–47.05)	53.74 (52.00–55.49)	65.94 (65.43–66.45)	41,314
Female household head only	27.53 (27.10–2.80)	41.82 (40.40–43.24)	34.95 (33.28–36.62)	23.82 (23.37–24.27)	16,256
Household head’s age	52.45 (52.27–52.63)	49.52 (48.97–50.06)	53.45 (52.72–54.18)	52.92 (52.72–53.12)	
Household size	2.56 (2.55–2.58)	2.82 (2.77–2.87)	2.77 (2.71–2.82)	2.49 (2.47–2.51)	

**Table 2:** Percentage of households with a member with one or multiple health outcomes, based on IRI MedProfiler 2015 data.

	All Incomes	Household (HH) Income < = 130% Poverty Income Group (IG)1	HH Income (130–185%] Poverty IG2	HH Income > 185% Poverty IG3	
Households with a member with:	Weighted percentage (95% CI)				33,179
Type 2 diabetes (T2D)	14.13 (13.69–14.58)	16.19 (14.73–17.64)	16.04 (14.39–17.70)	13.50 (13.02–13.97)	5945
Cardiovascular disease (CVD)	42.15 (41.45–42.85)	39.97 (37.88–42.06)	44.67 (42.15–47.19)	42.30 (41.53–43.07)	16,931
Obesity	21.48 (20.87–22.08)	26.90 (24.93–28.87)	23.36 (21.27–25.44)	20.17 (19.53–20.81)	7409
Smokes cigarettes	16.36 (15.82–16.89)	27.02 (25.06–28.98)	22.10 (20.00–24.20)	13.55 (13.03–14.07)	5587
T2D, CVD, and obesity	4.47 (4.22–4.72)	5.92 (5.08–6.75)	5.53 (4.56–6.50)	4.05 (3.79–4.32)	1906
No T2D, CVD, or obesity	46.60 (45.85–47.34)	45.01 (42.75–47.27)	43.28 (40.66–45.91)	47.30 (46.49–48.12)	12,939

**Table 3:** Adjusted weighted mean Healthy Eating Index (HEI)-2015 and component scores by household income level, based on IRI Consumer Network 2015 data.

	Mean (Standard Deviation [Std. Dev.])				Statistical Significance of Difference of Means between Income Groups *p*-Value		
Measure (Max Score)	All Incomes	Household (HH) Income < = 130% Poverty Income Group (IG)1	HH Income (130–185%] Poverty IG2	HH Income > 185% Poverty IG3	IG1 vs. IG2	IG1 vs. IG3	IG2 vs. IG3
HEI-2015 (100)	51.86 (1.84)	48.24 (1.10)	49.56 (1.07)	52.57 (1.13)	<0.001	<0.001	<0.001
Total vegetables (5)	2.07 (0.15)	1.89 (0.15)	1.98 (0.14)	2.11 (0.13)	<0.001	<0.001	<0.001
Greens and beans (5)	1.71 (0.19)	1.37 (0.12)	1.50 (0.09)	1.78 (0.14)	<0.001	<0.001	<0.001
Total fruits (5)	1.59 (0.16)	1.27 (0.08)	1.45 (0.10)	1.65 (0.11)	<0.001	<0.001	<0.001
Whole fruits (5)	1.69 (0.21)	1.27 (0.14)	1.45 (0.12)	1.76 (0.12)	<0.001	<0.001	<0.001
Whole grains (10)	3.42 (0.25)	2.91 (0.20)	3.14 (0.14)	3.51 (0.14)	<0.001	<0.001	<0.001
Total dairy (10)	5.39 (0.72)	5.15 (0.76)	5.41 (0.72)	5.42 (0.71)	<0.001	<0.001	0.58
Total protein foods (5)	3.09 (0.15)	2.90 (0.12)	3.00 (0.16)	3.12 (0.14)	<0.001	<0.001	<0.001
Seafood and plant proteins (5)	3.62 (0.30)	3.14 (0.26)	3.33 (0.22)	3.72 (0.23)	<0.001	<0.001	<0.001
Fatty acid (10)	4.85 (0.73)	5.04 (0.84)	4.88 (0.71)	4.83 (0.72)	<0.001	<0.001	<0.001
Sodium (10)	5.86 (0.19)	5.52 (0.18)	5.58 (0.25)	5.93 (0.14)	<0.001	<0.001	<0.001
Refined grains (10)	7.90 (0.43)	7.76 (0.41)	7.67 (0.49)	7.94 (0.43)	<0.001	<0.001	<0.001
Saturated fat (10)	5.77 (0.63)	6.04 (0.76)	5.84 (0.65)	5.73 (0.59)	<0.001	<0.001	<0.001
Added sugar (10)	4.89 (0.53)	3.98 (0.40)	4.34 (0.40)	5.07 (0.38)	<0.001	<0.001	<0.001

Note: Adjusted for household race and ethnicity; household head’s age and marital status; and female-led household. Unadjusted measures are omitted for brevity but can be obtained from the authors upon request.

**Table 4:** Adjusted weighted mean HEI-2015 and component scores (standard deviations) for households with and without a member with reported T2D or CVD, based on Consumer Network (CN) 2015-MedpProfiler data.

Measure (Max Score)	No Type 2 Diabetes	Type 2 Diabetes	% Difference	Difference between Groups *p*-Value	No CVD	CVD	% Difference	Difference between Groups *p*-Value
HEI-2015 (100)	52.41 (1.98)	51.19 (1.89)	2.38	<0.001	52.94 (2.16)	51.29 (1.92)	3.22	<0.001
Total vegetables (5)	2.09 (0.15)	2.12 (0.16)	−1.14	<0.001	2.10 (0.15)	2.09 (0.16)	0.46	<0.001
Greens and beans (5)	1.76 (0.21)	1.58 (0.17)	11.10	<0.001	1.80 (0.22)	1.64 (0.18)	9.39	<0.001
Total fruits (5)	1.62 (0.16)	1.38 (0.21)	17.40	<0.001	1.64 (0.16)	1.46 (0.24)	12.24	<0.001
Whole fruits (5)	1.72 (0.24)	1.57 (0.29)	9.30	<0.001	1.77 (0.24)	1.55 (0.30)	14.37	<0.001
Whole grains (10)	3.48 (0.25)	3.12 (0.34)	11.61	<0.001	3.59 (0.26)	3.18 (0.31)	12.81	<0.001
Total dairy (10)	5.45 (0.66)	5.24 (0.72)	3.97	<0.001	5.44 (0.67)	5.29 (0.67)	2.81	<0.001
Total protein foods (5)	3.10 (0.16)	3.22 (0.17)	−3.77	<0.001	3.14 (0.17)	3.09 (0.16)	1.63	<0.001
Seafood and plant proteins (5)	3.70 (0.30)	3.68 (0.32)	0.42	<0.001	3.76 (0.32)	3.62 (0.31)	3.80	0.03
Fatty acid (10)	4.79 (0.68)	5.11 (0.81)	−6.27	<0.001	4.81 (0.69)	4.94 (0.72)	−2.66	<0.001
Sodium (10)	5.99 (0.18)	5.34 (0.33)	12.04	<0.001	6.04 (0.25)	5.76 (0.16)	4.87	<0.001
Refined grains (10)	8.00 (0.42)	8.03 (0.30)	−0.45	<0.001	8.03 (0.45)	8.07 (0.31)	−0.40	<0.001
Saturated fat (10)	5.77 (0.57)	5.48 (0.76)	5.31	<0.001	5.81 (0.55)	5.70 (0.67)	1.93	<0.001
Added sugar (10)	4.94 (0.53)	5.30 (0.57)	−6.85	<0.001	5.03 (0.52)	4.91 (0.58)	2.35	0.01

Adjusted for household poverty level, size, race, and ethnicity; household head’s age and marital status; and female-led household. Note: Unadjusted measures are omitted for brevity but can be obtained from the authors upon request.

**Table 5:** Adjusted weighted mean HEI-2015 and component scores (standard deviations) for households with and without a member with reported obesity or the three conditions (T2D, CVD, and obesity), based on CN 2015-MedpProfiler data.

Measure (Max Score)	No Obesity	HH with Member (S) with Obesity	% Difference	Difference between Groups *p*-Value	HH with None of the Conditions	HH with Three Conditions	% Difference	Difference between Groups *p*-Value
HEI-2015 (100)	52.56 (1.97)	50.86 (1.83)	3.34	<0.001	53.29 (2.20)	50.23 (2.07)	6.10	<0.001
Total vegetables (5)	2.10 (0.15)	2.08 (0.14)	1.00	<0.001	2.09 (0.15)	2.04 (0.16)	2.34	0.02
Greens and beans (5)	1.76 (0.21)	1.64 (0.18)	7.11	<0.001	1.81 (0.22)	1.46 (0.17)	24.20	<0.001
Total fruits (5)	1.63 (0.17)	1.43 (0.14)	14.13	<0.001	1.69 (0.18)	1.32 (0.22)	28.05	<0.001
Whole fruits (5)	1.73 (0.24)	1.58 (0.22)	9.67	<0.001	1.80 (0.24)	1.52 (0.26)	18.90	<0.001
Whole grains (10)	3.50 (0.25)	3.14 (0.27)	11.48	<0.001	3.67 (0.25)	2.97 (0.40)	23.29	<0.001
Total dairy (10)	5.41 (0.68)	5.41 (0.68)	−0.03	0.003	5.42 (0.67)	5.30 (0.70)	2.34	<0.001
Total protein foods (5)	3.10 (0.16)	3.18 (0.16)	−2.43	<0.001	3.12 (0.17)	3.14 (0.22)	−0.84	<0.001
Seafood and plant proteins (5)	3.70 (0.30)	3.68 (0.30)	0.47	<0.001	3.76 (0.31)	3.58 (0.32)	4.99	<0.001
Fatty acid (10)	4.89 (0.70)	4.66 (0.75)	4.86	<0.001	4.87 (0.70)	4.89 (0.83)	−0.37	<0.001
Sodium (10)	5.95 (0.16)	5.63 (0.30)	5.72	<0.001	6.11 (0.25)	5.33 (0.44)	14.64	<0.001
Refined grains (10)	7.97 (0.40)	8.09 (0.40)	−1.43	<0.001	8.01 (0.47)	8.12 (0.37)	−1.40	<0.001
Saturated fat (10)	5.79 (0.59)	5.41 (0.67)	7.18	<0.001	5.92 (0.53)	5.39 (0.87)	9.79	<0.001
Added sugar (10)	5.02 (0.55)	4.94 (0.51)	1.77	<0.001	5.02 (0.52)	5.16 (0.57)	−2.67	<0.001

Adjusted for household poverty level, size, race, and ethnicity; household head’s age and marital status; and female-led household. Note: Unadjusted measures are omitted for brevity but can be obtained from the authors upon request.

**Table 6:** Adjusted weighted mean HEI-2015 and component scores (standard deviations) for households with and without a member reporting smoking, based on CN 2015-MedpProfiler data.

Measure (Max Score)	No Smoker in HH	HH with Smoker(s)	% Difference	Difference between Groups *p*-Value
HEI-2015 (100)	52.95 (1.83)	47.91 (1.34)	10.52	<0.001
Total vegetables (5)	2.11 (0.16)	2.01 (0.13)	5.25	<0.001
Greens and beans (5)	1.79 (0.19)	1.42 (0.15)	26.24	<0.001
Total fruits (5)	1.67 (0.15)	1.09 (0.14)	53.42	<0.001
Whole fruits (5)	1.80 (0.23)	1.13 (0.17)	59.56	<0.001
Whole grains (10)	3.59 (0.24)	2.51 (0.14)	43.32	<0.001
Total dairy (10)	5.47 (0.68)	5.12 (0.68)	6.81	<0.001
Total protein foods (5)	3.15 (0.16)	2.95 (0.16)	6.59	<0.001
Seafood and plant proteins (5)	3.76 (0.28)	3.35 (0.32)	12.10	<0.001
Fatty acid (10)	4.85 (0.70)	4.78 (0.79)	1.46	<0.001
Sodium (10)	5.91 (0.18)	5.69 (0.29)	3.88	<0.001
Refined grains (10)	7.98 (0.41)	8.08 (0.40)	−1.19	0.002
Saturated fat (10)	5.74 (0.59)	5.51 (0.73)	4.14	<0.001
Added sugar (10)	5.14 (0.51)	4.29 (0.50)	19.88	<0.001

Note: Adjusted for household poverty level, size, race, and ethnicity; household head’s age and marital status; and female-led household. Unadjusted measures are omitted for brevity but can be obtained from the authors upon request.

## Data Availability

Not applicable.
